# Clenbuterol Abuse in Bodybuilding and Athletics: Unsupervised Use, Psychological Motivations, and Health Consequences

**DOI:** 10.7759/cureus.84904

**Published:** 2025-05-27

**Authors:** Suhas Kataveni, Raga Priya Gourishetty, Shruti M Mundada, Moksha Prasoona Avvaru, Vijay S Kollipara, Sai Pranay Gottimukkala

**Affiliations:** 1 Internal Medicine, Gandhi Medical College and Hospital, Hyderabad, IND; 2 General Medicine, Srikara Hospitals, Hyderabad, IND

**Keywords:** anabolic agents, athletes, bodybuilding, clenbuterol, doping, performance-enhancing drugs, β2-adrenergic agonist

## Abstract

Clenbuterol is a β2-adrenergic agonist originally developed as a bronchodilator but is now often used without supervision for its perceived muscle-building and fat-burning effects. Although it is not approved for human use in many regions, it continues to be used in bodybuilding and athletics for physique and performance enhancement. Its appeal lies in its ability to influence muscle development and fat metabolism. Many individuals use it due to psychological motivators such as body image concerns and a desire for rapid physical changes. However, its use may result in side effects involving the heart, muscles, and overall physical well-being, such as arrhythmias, hypertension, and muscle spasms. In some cases, it has also been linked to accidental exposure through contaminated food. The widespread misuse of clenbuterol raises ethical concerns in competitive environments. More discussion is needed around developing safer methods of achieving similar goals, and there is a need for increased public awareness and caution regarding its use.

## Introduction and background

Clenbuterol is a long-acting β2-adrenergic agonist that was originally developed as a bronchodilator for the treatment of asthma in 1977; however, it has garnered attention for its off-label use in humans because of its potent anabolic and lipolytic effects [1]. Although not approved for human use in the USA and many European countries, it remains widely abused in the domains of bodybuilding, weight loss, and performance enhancement [2]. In skeletal muscle, clenbuterol activates β2-adrenergic receptors, which can stimulate hypertrophy through pathways involving the phosphatidylinositol 3-kinase/protein kinase B/mammalian target of rapamycin (PI3K/Akt/mTOR) axis and elevate the resting metabolic rate and fat oxidation [1].

With an increased focus on physical appearance in society, shortcut alternatives to dieting have been promoted. This has led to the increased consumption of a range of "image- and performance-enhancing drugs," which can include β2 agonists such as clenbuterol and salbutamol [3]. Clenbuterol can also act on β3-adrenergic receptors on adipocytes, promoting weight loss and increasing muscle mass. A major problem lies among elite, amateur athletes and bodybuilders, who aim to enhance their physical performance and improve their appearance while reducing their body fat [3].

Apart from this intentional intake of clenbuterol, there have been instances of supplements labeled as thermogenic products, metabolism boosters, and adulterated with small amounts of clenbuterol, which were marketed as fat burners. The Food and Drug Administration (FDA) and health authorities in multiple countries have issued warnings about clenbuterol being found in dietary products such as "fat burners" sold under names such as ClenXDV, Clenbuterall, or Clen 40; herbal mixes; or anabolic cocktails [4-6]. Recently, another source of inadvertent doping has been found to be the consumption of contaminated meat. The anabolic effect of clenbuterol promotes the growth of cattle for the production of lean meat. As a result, athletes who consume such contaminated meat may unknowingly ingest trace amounts of clenbuterol, potentially leading to an adverse analytical finding (AAF), which is a positive anti-doping test result indicating the presence of a banned substance in their biological sample [7]. If confirmed, the athlete may face provisional suspension, sanctions, or disqualification. Testing is conducted in World Anti-Doping Agency (WADA)-accredited laboratories, and even unintentional ingestion must be proven by the athlete [4]. Differentiating between unintentional consumption and clenbuterol doping remains challenging [7,8].

The pharmacodynamic actions of clenbuterol have also made it a possible candidate for mitigating muscle atrophy in clinical conditions, such as spinal cord injury, where traditional anabolic agents often show limited efficacy [9]. Therapeutic clenbuterol, mainly for asthma, can be prescribed in the dosage range of 20-40 µg daily, whereas a peak "fat-burning" dose is typically in the range of 120-160 µg daily [3]. Despite its therapeutic potential, clenbuterol is associated with significant risks, especially at high doses. Human toxicity has been associated with sympathomimetic side effects, such as tachycardia, tremors, hypokalemia, and cardiac arrhythmias. These dangers, coupled with increasing cases of misuse and contamination, have raised global concerns regarding their regulations and safety profiles [10].

## Review

Pharmacology

Clenbuterol is a β2 sympathomimetic bronchodilator primarily used to reduce peripheral airway obstruction in bronchial asthma. The oral administration of clenbuterol (40 μg) significantly increased the maximal flow in the effort-independent phase of both maximal expiratory flow volume (MEFV) and partial expiratory flow volume (PEFV) curves and markedly decreased the frequency dependence of dynamic compliance in comparison with the baseline values, while it improved both vital capacity (VC) and forced expiratory volume in one second (FEV1) to a lesser extent [11]. It is a long-acting, oral β2-adrenergic partial agonist that stimulates receptors to relax bronchial smooth muscle, inhibit the release of inflammatory mediators, and decrease mucus production and may increase the rate of mucociliary transport in the airways, thus helping in the treatment of acute asthma [2]. Apart from these effects, clenbuterol also increases muscle mass and decreases body fat, prompting its abuse among athletes and bodybuilders [2].

Anabolic Effects and Lipolysis

Clenbuterol stimulates cyclic adenosine monophosphate (cAMP) levels and suppresses fasting-induced expression of atrogin 1 and muscle RING-finger protein-1 (MuRF1) by inducing Akt/forkhead box O3 (FoxO3) phosphorylation. It also regulates the muscle wasting process through the activation of protein anabolism and the inhibition of cathepsin L and ubiquitin ligase-dependent proteolytic system [12].

**Figure 1:**
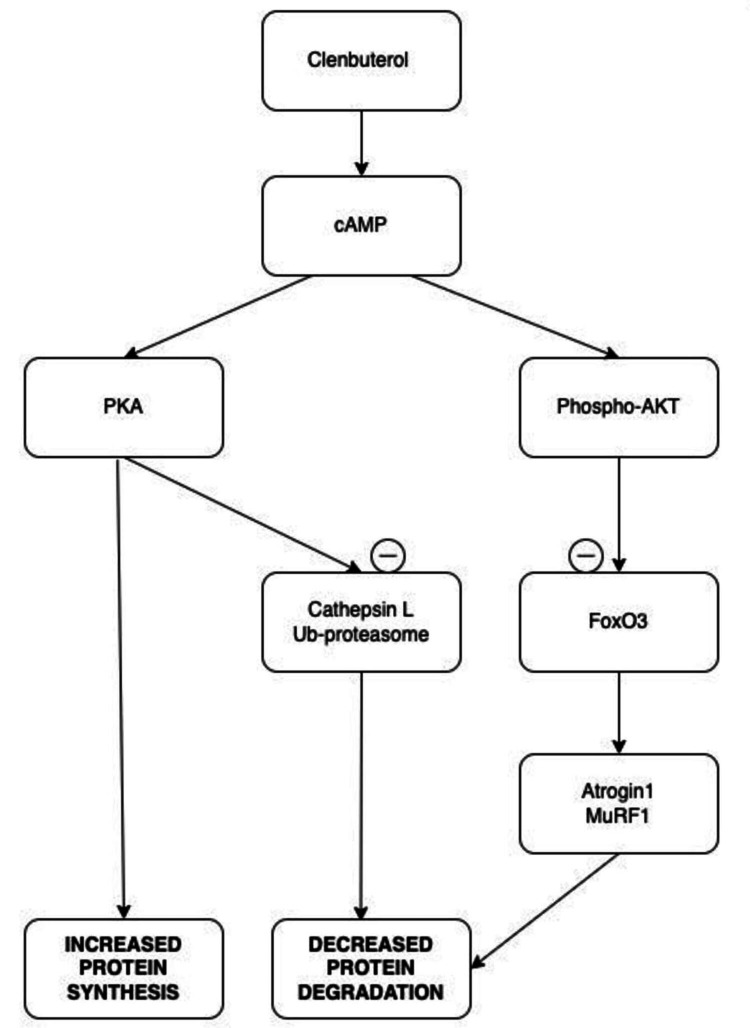
Action of clenbuterol (catabolic and anabolic). Created by the authors based on data from Dutt et al. [12]. cAMP, cyclic adenosine monophosphate; PKA, protein kinase A; Phospho-AKT, phosphorylated protein kinase B; Ub-proteasome, ubiquitin-proteasome; FoxO3, forkhead box O3 (a transcription factor); MuRF1, muscle RING-finger protein-1 (another E3 ubiquitin ligase)

In young healthy men, clenbuterol ingestion significantly influences various metabolic parameters [1]. It increases the resting metabolic rate by 21% and enhances fat oxidation by 39%. At the molecular level, clenbuterol elevates the phosphorylation of mTOR at Ser2448 by 121% and protein kinase A (PKA) substrates by 35%, indicating enhanced anabolic and adrenergic signaling. Despite these molecular and metabolic shifts, skeletal muscle glycogen content remains unchanged. Clenbuterol also induces marked increases in plasma concentrations of several substrates and hormones, including glucose (25%), lactate (87%), insulin (105%), and free fatty acids (129%), reflecting widespread systemic metabolic activation [1]. These results imply that clenbuterol, in the short term, promotes fat loss by enhancing resting metabolic rate and fat oxidation while simultaneously activating anabolic pathways such as mechanistic target of rapamycin (mTOR) and protein kinase A (PKA) that support muscle protein synthesis. These combined effects contribute to its widespread use as a performance-enhancing agent. However, over the long term, the significant increases in plasma glucose, insulin, and lactate levels raise concerns about potential metabolic strain. Prolonged or high-dose use may predispose individuals to insulin resistance, glycemic dysregulation, and increased cardiovascular risk, highlighting the dangers associated with its misuse or chronic exposure (Figure 1).

Pharmacokinetics

In a study by Yamamoto et al. in 1985, therapeutic doses of clenbuterol hydrochloride were administered to men at 20 μg, 40 μg, and 80 μg. The unmetabolized drug in plasma and urine was determined using an enzyme immunoassay [13].

Following the oral administration of clenbuterol hydrochloride at doses of 20 μg, 40 μg, and 80 μg, peak plasma concentrations were observed within 2-3 hours, reaching 0.1 ng/mL, 0.2 ng/mL, and 0.35 ng/mL, respectively. The peak levels rose in a dose-dependent manner within approximately 2.5 hours, and the pharmacological effects persisted for about six hours. Clenbuterol exhibits a relatively long half-life of approximately 35 hours, which contributes to its sustained pharmacological effects, including prolonged lipolysis and bronchodilation. In terms of distribution, after a single 80 μg dose, 96%-98% of the drug was bound to plasma proteins over a 24-hour period. With repeated dosing, a steady-state plasma concentration was achieved by the fourth day; levels stabilized between 0.2 and 0.3 ng/mL with twice-daily 20 μg doses and between 0.5 and 0.6 ng/mL with twice-daily 40 μg doses. Regarding elimination, approximately 20% of the administered clenbuterol was excreted via the urine within 72 hours, primarily in unchanged form or as glucuronide and sulfate conjugates [13].

Use in bodybuilding and athletics

Experiments have suggested significant increases in gastrocnemius muscle mass, protein, and RNA content and a decrease in epididymal fat pad mass caused by clenbuterol, which has become a popular anabolic substitute for steroids among the community of bodybuilders and athletes [14]. The prevalence of clenbuterol abuse or the use of other beta-agonists is not well documented. According to the World Anti-Doping Agency (WADA) in 2022, β2 agonists accounted for 3% of all adverse analytical findings (AAFs), among which clenbuterol accounted for 37%.

The recommended adult human dose of clenbuterol for therapeutic use in asthma is 20-40 μg twice daily orally or 20 μg by inhalation eight-hourly [15]. The range of the anabolic dose of clenbuterol in athletes is 60-120 μg daily for 6-12 weeks [16]. The anabolic effects on muscles cannot be sustained by the chronic use of clenbuterol, primarily due to β2-adrenoceptor (β2-AR) desensitization. Prolonged stimulation leads to receptor downregulation and the diminished activation of downstream signaling pathways. Although clenbuterol acutely increases blood flow to the muscles, it significantly reduces blood flow when used chronically. This response is associated with decreased skeletal muscle beta-adrenoceptor density [15].

According to a report by Prather et al. in 1995, athletes have reported using clenbuterol for its anabolic and lipolytic effects [17]. The most common route of intake is oral in the form of tablets or capsules. A schedule of three weeks on and three off was utilized. During the three-week "on" period, the drug is taken for two consecutive days and discontinued for two days, presumably to avoid some of the receptor downregulation mentioned [17].

Athletes who have been using anabolic steroids and must present to competition with clean urine have to discontinue the usage of steroids well in advance of contest dates. Clenbuterol is used following the discontinuation of steroids to retard muscle mass loss and for improved muscle definition [17].

Adverse effects of clenbuterol use

Clenbuterol, a β2-adrenergic agonist, is a potent sympathetic stimulant. While it is sometimes misused owing to its anabolic and lipolytic effects, its adverse effects can be severe and multisystemic (Table 1) [18,19].

**Table 1:** Adverse effects of clenbuterol. CK-MB: creatine kinase-myocardial band

Common Side Effects	Severe/Toxic Effects
Arrhythmias	Supraventricular tachycardia
Hypertension	Pulmonary edema
Palpitations	Myocardial infarction
Muscle spasms	Elevated cardiac biomarkers (troponin and CK-MB)
Hyperreflexia	Electrocardiographic changes (e.g., ST elevations)
Headache	Wide pulse pressure
Agitation	Rhabdomyolysis
Nausea or vomiting	Lactic acidosis
Hypokalemia	Seizures
Mydriasis	Psychosis

A descriptive study of drug abuse reported that clenbuterol is frequently misused alongside other performance-enhancing substances, which may potentiate its cardiovascular and neurological toxicity [18]. This polypharmacy complicates the identification of the primary causative agent in adverse events.

Nonetheless, multiple case reports have documented cardiovascular toxicity, including myocardial infarction (MI) and arrhythmias, attributable solely to clenbuterol, reinforcing its potential for harm even when used alone [20-23].

Unsupervised utilization and its drivers

Bodybuilders display obsessive-compulsive disorder, behavioral addiction, mental health problems, and a body dysmorphic disorder/body image disorder known as muscle dysmorphia or bigorexia [24]. The use of anabolic steroids and supplements arises from dissatisfaction with oneself. Unsurprisingly, there is a high prevalence of doping (77.8%) among competitive bodybuilders [24]. Androgenic anabolic steroid users may have a fascination with the effects of pharmacological substances on human physiology, be motivated by a desire for well-being and to look and feel good, want to enjoy life fully even if that entails taking risks, and want to "prepare for and perform at competitions" [24]. Their investment both in and outside the gym is rather the expression of their lifestyles, expertise, performance, etc. The unsupervised utilization of clenbuterol among athletes and bodybuilders is a growing concern, driven by a desire to enhance physical appearance and performance without medical oversight. Clenbuterol, originally developed as a bronchodilator, has gained popularity in the bodybuilding community owing to its ability to reduce fat and increase lean muscle mass. Despite its potential for severe adverse effects, athletes continue to use clenbuterol because of its perceived effectiveness and rapid results [25].

Psychological and Social Drivers

Desire for an "extraordinary self": One of the primary motivations for using unsupervised clenbuterol is the pursuit of an idealized, "extraordinary" version of oneself. Bodybuilders often perceive clenbuterol as a means of rapidly achieving a competitive physique, aligning with their identity as high-performing athletes [25]. This desire to transform physically while maintaining a sense of normalcy outside the gym creates conflict between the "extraordinary" and "ordinary" selves.

Balancing athletic aspirations and everyday life: Athletes may rationalize the use of clenbuterol as a necessary step to maintain both their athletic goals and personal lives. As described in the context of other appearance and performance-enhancing drugs (APEDs), individuals often perceive the use of performance enhancers as essential for maintaining both their competitive edge and social functionality [25].

Risk Perception and User Typology

According to Christiansen et al., users of APEDs, including clenbuterol, can be classified into distinct types based on their perception of risk and focus on effectiveness [26].

High effectiveness/low risk (scientific approach): Users in this category meticulously research the best ways to maximize benefits while minimizing harm.

Desire for immediate self-enhancement type (risk-accepting): These users are less concerned with potential health consequences and motivated by short-term gains.

Well-being type (moderate use): Individuals in this group use clenbuterol in controlled doses, believing that it helps maintain health while enhancing performance.

Athlete type (competition-oriented): For this group, clenbuterol use was linked primarily to achieving competitive success.

Evolution Over Time

It is noteworthy that users may transition between these types throughout their athletic career. Early-stage users might adopt a risk-accepting approach, while more experienced athletes often shift toward a more calculated and health-conscious perspective as they recognize long-term health risks [25].

Influence of Social and Cultural Factors

Peer influence and social norms: The normalization of clenbuterol use within bodybuilding communities significantly influences unsupervised utilization. Athletes who see peers using the substance without apparent immediate consequences may perceive the risks as manageable or overstated [25].

Societal expectations and media influence: Social media platforms and online fitness communities often portray clenbuterol as a harmless and effective shortcut to the ideal physique. This positive framing can overshadow scientific evidence highlighting the health risks associated with unsupervised use [27].

Ambiguity in risk perception: Users often perceive themselves as sufficiently knowledgeable to manage the risks associated with clenbuterol. However, this sense of control can lead to a dangerous underestimation of potential adverse effects, including cardiovascular and neurological complications [25].

Clinical trials in animals

Clenbuterol is a β2 agonist used as a bronchodilator to treat bronchospasm in horses. It is known for its muscle-specific anabolic response in lambs, broiler chickens, rats, horses, and steers.

Synopsis of Cardiovascular Effects of Clenbuterol in Animals

Clenbuterol, a β2-adrenergic agonist, has been associated with several adverse cardiovascular effects.

Cardiac hypertrophy and collagen infiltration: The chronic administration of clenbuterol (1.5-2 mg/kg) induces cardiac hypertrophy and collagen infiltration around the blood vessels and within the left ventricular wall in rodents [28].

Myocyte necrosis and oxygen supply reduction: Prolonged clenbuterol use (0.003-3 mmol/kg for 14 days) resulted in cardiac and skeletal muscle necrosis in rats, potentially due to reduced oxygen supply and increased muscle fatigability [29].

Adverse cardiac changes in horses: In equine studies, long-term clenbuterol administration increased the heart rate (HR), right ventricular pressure (RVP), and aortic root diameter after maximal exercise, raising the risk of aortic root rupture. Additionally, increased drug concentration (4-12-fold) in cardiac and skeletal myocytes was noted [30].

Muscle apoptosis: Studies have demonstrated cardiac and skeletal muscle apoptosis in rats, emphasizing the potential long-term risks of clenbuterol use [29].

Synopsis of the Effects of Clenbuterol on the Skeletal Muscles of Animals

Shifts in muscle fiber types: Clenbuterol in rats caused a transformation from slow-twitch (aerobic) to fast-twitch (anaerobic) fibers, which may be detrimental to endurance performance. This shift results in muscles becoming more suited for short bursts of activity rather than for sustained endurance [31,32].

Altered muscle contractility: Clenbuterol increased the cross-sectional area (CSA) of muscles, leading to increased muscle mass and tension. However, it also causes a decrease in fatigue resistance and reduces muscle endurance capacity [31].

Reduced aerobic performance: Clenbuterol reduced the proportion of type IIA muscle fibers, which are intermediate and have aerobic potential, replacing them with more type IIx fibers, which are less suited for aerobic exercise. This shift compromises endurance and aerobic performance [33,34].

Calcium sensitivity changes: Clenbuterol alters calcium sensitivity in fast-twitch fibers, which can affect muscle contraction efficiency, especially during prolonged activities [35,36].

Potential for decreased exercise performance: In studies with animals (e.g., horses), clenbuterol use was associated with decreased aerobic performance, likely due to the altered muscle composition and increased muscle mass, which are less favorable for endurance tasks [33,34].

Synopsis of the Effects of Clenbuterol on Fat Mass

Reduction in fat mass: Clenbuterol significantly reduces fat mass, with studies showing a decrease in fat percentage in horses treated with different doses of clenbuterol. One study found a continual decrease in fat with a higher dose (3.2 µg/kg) compared with a lower dose (2.4 µg/kg) administered more frequently [37].

Increase in fat-free mass (FFM): Clenbuterol increases fat-free mass (FFM) in horses both with and without exercise. The increase was more rapid in the clenbuterol-only group, becoming significant after two weeks, compared to the four weeks needed in the clenbuterol-plus-exercise group. This suggests that clenbuterol and exercise might act synergistically for fat loss but antagonistically for FFM gain [37].

Altered adipokine levels: Clenbuterol administration significantly increased adiponectin and decreased leptin levels in horses. Both adipokines are related to fat mass, and these changes correlate with fat reduction. These alterations indicate that clenbuterol not only reduces fat but also affects the hormonal regulation of appetite and energy balance [38].

Human studies and case reports

No human studies have been conducted on non-asthmatic adults. The FDA and Food Safety and Inspection Service (FSIS) have banned this drug for human consumption. Clenbuterol has not been studied in non-asthmatic adults due to serious health concerns related to its adverse effects on the cardiovascular system, such as increased heart rate, hypertension, and arrhythmias, which can be dangerous for individuals without respiratory conditions such as asthma [39]. It is used in animals to enhance their performance and muscle build. The FDA and FSIS banned this drug for performance and muscle build in both food and animals.

A randomized clinical trial of clenbuterol in spinal and bulbar muscular atrophy showed modest improvement in handgrip and creatine kinase levels in the blood but no overall improvement in walking and respiratory function [40]. There was also an increase in tremors and heart rate in the treatment group.

Clenbuterol has shown potential in preclinical models of cancer cachexia management in combating muscle loss, but safety concerns prevent its use in human trials [41]. It may be effective in preventing muscle loss by cachexia in animal models, but it is not approved for human studies because of potential side effects and serious cardiovascular risks [42].

In a study on skeletal muscle gene expression by clenbuterol, extrapolated measurements suggested that clenbuterol can increase daily energy expenditure from approximately 2000 to 2400 kcal [1]. However, such extrapolation assumes a sustained ~20% increase over 24 hours, which may not reflect the actual pharmacodynamics, as the effect of clenbuterol likely diminishes within that period.

Nevertheless, it is plausible that the elevated metabolic rate persists longer than that with other β2 agonists, such as formoterol or salbutamol, due to the longer elimination half-life of clenbuterol (~25-35 hours versus ~12 hours for formoterol and ~3-4 hours for salbutamol) [1]. This thermogenic effect likely contributes to fat mass reduction, as demonstrated in rodent studies, and is widely claimed within bodybuilding circles.

Skeletal muscles are densely populated with β2-adrenoceptors (β2-AR), which are G-protein-coupled receptors. β2-AR signaling plays a critical role in regulating both skeletal muscle proliferation and differentiation, making it an attractive therapeutic target for managing skeletal muscle wasting conditions. Several β2-adrenoceptor agonists, including clenbuterol and formoterol, are currently being investigated for their potential to mitigate skeletal muscle atrophy through the modulation of this signaling pathway [12].

In a study on the use of adrenergic agonists in treating urinary incontinence, clinical trials were conducted using clenbuterol with other adrenergic agonists (such as phenylpropanolamine, norepinephrine, and midodrine) to analyze general outcomes [43]. The overall evidence suggests weak benefits of adrenergic agonists (as a class) in improving subjective symptoms, such as reduced incontinence episodes and pad usage, but not achieving full continence. Side effects (such as insomnia, restlessness, and vasomotor symptoms) occurred in 28% of participants on adrenergic drugs but were generally minor, with few leading to dropouts. However, because the average number of participants per trial was only 42, the strength and applicability of the results should be interpreted with caution [43].

Clenbuterol has novel therapeutic potential beyond its anabolic and thermogenic effects in the management of neuropathic pain [44]. Its mechanism of action mimics that of antidepressants, which also require chronic administration for its efficacy in neuropathic pain management. Pain management persisted across various delivery methods (inhalation, implant, and transdermal gel) [44].

Various cases have been reported showing the negative effects of clenbuterol on patients. A case report showed a young bodybuilder patient in his 20s, who is a frequent user of clenbuterol for bodybuilding, who presented with acute chest pain. It was later diagnosed as MI with ST-segment elevation on ECG, with elevated serum troponin levels and grade 4 thrombus in the proximal left anterior descending artery [45].

A case report showed a patient who was on low-dose clenbuterol (40 μg/day) for one week and presented with complaints of chest pain for three days [22]. ECG showed ST depression and elevated troponin levels. An initial provisional diagnosis of type II MI was considered. Cardiac MRI showed delayed epicardial enhancement and cardiac edema, consistent with myocarditis. This case clarified that even standard or low doses of clenbuterol (40 μg/day) can cause serious adverse cardiac events.

Food contamination

Food safety remains a critical concern in global public health, particularly when it involves contamination with pharmacological agents that are not approved for human consumption. One such agent is clenbuterol, a β2-adrenergic agonist that has drawn increasing attention owing to its illegal use in animal agriculture. Originally developed and approved for veterinary use as a bronchodilator, clenbuterol has been used as a growth promoter in livestock to enhance lean muscle mass and reduce the fat content. This misuse poses substantial health risks to consumers and has been linked to numerous foodborne toxicity outbreaks across multiple countries.

In several countries, such as China and Mexico, the sympathomimetic and anabolic agent clenbuterol has been illegally used as a growth promoter in animal production [8]. Specifically, clenbuterol increases muscle mass while simultaneously decreasing fat mass [46]. As a repartitioning agent, horses treated with clenbuterol at 2.4 μg/kg twice daily (one group treated with clenbuterol and exercise and one group treated with just clenbuterol) lost 20 kg and 15 kg of fat mass, respectively, after two weeks [46]. These properties have prompted their illicit use in cattle, pigs, and other meat-producing animals in several countries, despite widespread bans and strict regulations.

The presence of clenbuterol in meat products has caused several outbreaks of toxicity in humans.

People who have consumed clenbuterol-contaminated meat have demonstrated symptoms of drug toxicity, including skeletal muscle tremors, tachycardia, cephalalgia, myalgia, nervousness, dizziness, and nausea [47]. In Spain, outbreaks were reported after the ingestion of a cow's liver, with roughly 300 cases reported between 1989 and 1992 [47].

Similar outbreaks were seen in France, resulting in the incidence of food poisoning in 22 cases by residues of clenbuterol in the veal liver in 1990 [4], and around a total of 2455 cases by the year 2025 were seen in China [48]. Clenbuterol contamination in food products highlights the intersection of veterinary pharmacology, agricultural practices, and public health risks. While its pharmacological effects may offer economic advantages in livestock production, the unintended consequences for human health are severe and well documented. Continued vigilance, improved regulatory frameworks, and public awareness are essential to mitigate the threat posed by pharmacological contaminants in the global food supply chain.

Legal and ethical concerns

The use of clenbuterol as a performance-enhancing drug raises profound legal and ethical concerns in professional and amateur sports, as well as its broader implications for public trust in competitive athletics. Clenbuterol is classified as a prohibited substance by the World Anti-Doping Agency (WADA) because of its anabolic and performance-enhancing properties, which include increased lean muscle mass, enhanced aerobic capacity, and accelerated fat metabolism. Similarly, the US Food and Drug Administration (FDA) prohibits the use of clenbuterol in food-producing animals and lists it as an unapproved substance for human use outside of specific veterinary applications.

In routine sports drug testing, clenbuterol can be detected in urine at concentrations of a few pg/mL using liquid chromatography-mass spectrometry approaches [8]. Effective June 1, 2019, a new amendment was introduced by WADA stating that low levels of clenbuterol (urine concentrations of <5 ng/mL) present in an athlete's sample can be reported by a WADA-accredited laboratory as an atypical finding rather than an automatic violation. In such instances, further investigation is conducted to assess the plausibility of meat contamination as a source. Conversely, concentrations of ≥5 ng/mL are processed through the standard anti-doping adjudication pathway, with potential sanctions depending on the context and history of the athlete [49].

Notable legal cases and precedents

A notable example is the case of Alberto Contador, the Spanish cyclist and the three-time Tour de France winner, who tested positive for clenbuterol in 2010. He claimed that the result was due to the consumption of contaminated meat during a race. Despite this defense, the Court of Arbitration for Sport (CAS) ruled against him in 2012, imposing a two-year ban and stripping him of his titles [50]. The American swimmer Jessica Hardy tested positive in US trials in 2008. She was subjected to a one-year suspension, which claimed that she unknowingly took the drug as a contaminated supplement [51]. In 2013, Rogers tested positive for clenbuterol after winning the Japanese Cup. He attributed this result to the consumption of contaminated meat during the Tour of Beijing [51]. Many such incidents have been documented over the years, and they provide users with an unjust competitive advantage by promoting lean muscle growth and fat loss with increased endurance. Unintentional doping due to the consumption of tainted meat has had not only physiological consequences but also reputational and professional damage for many athletes as seen above.

## Conclusions

Initially created for veterinary use, clenbuterol has become known for its off-label use in both animal and human contexts, particularly among fitness-focused individuals. Despite regulatory barriers in many countries, its popularity persists due to various personal and social pressures. These include concerns about physical appearance, peer influence, and the desire to improve performance or body image. Unmonitored use can carry significant risks, especially to cardiovascular and muscular health. Tackling this issue involves better public understanding, more careful food monitoring, and clearer differentiation between accidental and intentional exposure in performance settings. Promoting a culture of safety and responsible performance improvement is essential. Continued exploration into safer alternatives may offer future solutions.

## References

[1] Jessen S, Solheim SA, Jacobson GA, Eibye K, Bangsbo J, Nordsborg NB, Hostrup M (2020). Beta(2) -adrenergic agonist clenbuterol increases energy expenditure and fat oxidation, and induces mTOR phosphorylation in skeletal muscle of young healthy men. Drug Test Anal.

[2] Kintz P, Gheddar L, Ameline A, Dumestre-Toulet V, Verschoore M, Comte J, Raul JS (2019). Complete post-mortem investigations in a death involving clenbuterol after long-term abuse. J Anal Toxicol.

[3] Milano G, Chiappini S, Mattioli F, Martelli A, Schifano F (2018). Β-2 agonists as misusing drugs? Assessment of both clenbuterol- and salbutamol-related European Medicines Agency pharmacovigilance database reports. Basic Clin Pharmacol Toxicol.

[4] Geyer H, Parr MK, Mareck U, Reinhart U, Schrader Y, Schänzer W (2004). Analysis of non-hormonal nutritional supplements for anabolic-androgenic steroids - results of an international study. Int J Sports Med.

[5] Tucker J, Fischer T, Upjohn L, Mazzera D, Kumar M (2018). Unapproved pharmaceutical ingredients included in dietary supplements associated with US Food and Drug Administration warnings. JAMA Netw Open.

[6] (2025). Tainted weight loss products. https://www.fda.gov/drugs/medication-health-fraud/tainted-weight-loss-products.

[7] Geyer H, Schänzer W, Thevis M (2014). Anabolic agents: recent strategies for their detection and protection from inadvertent doping. Br J Sports Med.

[8] Walpurgis K, Thomas A, Geyer H, Mareck U, Thevis M (2020). Dietary supplement and food contaminations and their implications for doping controls. Foods.

[9] Otzel DM, Kok HJ, Graham ZA, Barton ER, Yarrow JF (2021). Pharmacologic approaches to prevent skeletal muscle atrophy after spinal cord injury. Curr Opin Pharmacol.

[10] Brett J, Dawson AH, Brown JA (2014). Clenbuterol toxicity: a NSW poisons information centre experience. Med J Aust.

[11] Hida W, Sakurai M, Ichinose M (1985). Effect of clenbuterol on peripheral airway obstruction in bronchial asthma. Curr Med Res Opin.

[12] Dutt V, Gupta S, Dabur R, Injeti E, Mittal A (2015). Skeletal muscle atrophy: potential therapeutic agents and their mechanisms of action. Pharmacol Res.

[13] Yamamoto I, Iwata K, Nakashima M (1985). Pharmacokinetics of plasma and urine clenbuterol in man, rat, and rabbit. J Pharmacobiodyn.

[14] Choo JJ, Horan MA, Little RA, Rothwell NJ (1992). Anabolic effects of clenbuterol on skeletal muscle are mediated by beta 2-adrenoceptor activation. Am J Physiol.

[15] Rothwell NJ, Stock MJ, Sudera DK (1987). Changes in tissue blood flow and beta-receptor density of skeletal muscle in rats treated with the beta2-adrenoceptor agonist clenbuterol. Br J Pharmacol.

[16] Barceloux DG (2012). Medical Toxicology of Drug Abuse: Synthesized Chemicals and Psychoactive Plants. Wiley.

[17] Prather ID, Brown DE, North P, Wilson JR (1995). Clenbuterol: a substitute for anabolic steroids?. Med Sci Sports Exerc.

[18] Gauthier J (2001). Doping in sports: cardiovascular effects. Ann Cardiol Angéiologie.

[19] Spiller HA, James KJ, Scholzen S, Borys DJ (2013). A descriptive study of adverse events from clenbuterol misuse and abuse for weight loss and bodybuilding. Subst Abus.

[20] Barry AR, Graham MM (2013). Case report and review of clenbuterol cardiac toxicity. J Cardiol Cases.

[21] Shafrir A, W Leibowitz DW, Alcalai R, Elitzur Y, Muszkat M (2019). Myocardial injury induced by the long acting beta2 adrenergic agonist clenbuterol. Cardiol Cardiovasc Med.

[22] Moriarty N, Attar N (2020). Clenbuterol-induced myocarditis: a case report. Eur J Case Rep Intern Med.

[23] Quinley KE, Chen HY, Yang HS, Lynch KL, Olson KR (2016). Clenbuterol causing non-ST-segment elevation myocardial infarction in a teenage female desiring to lose weight: case and brief literature review. Am J Emerg Med.

[24] Coquet R, Roussel P, Ohl F (2018). Understanding the paths to appearance- and performance-enhancing drug use in bodybuilding. Front Psychol.

[25] Macho J, Mudrak J, Slepicka P (2021). Enhancing the self: amateur bodybuilders making sense of experiences with appearance and performance-enhancing drugs. Front Psychol.

[26] Christiansen AV, Vinther AS, Liokaftos D (2017). Outline of a typology of men’s use of anabolic androgenic steroids in fitness and strength training environments*. Drugs Educ Prev Policy.

[27] Petróczi A, Aidman E (2008). Psychological drivers in doping: the life-cycle model of performance enhancement. Subst Abuse Treat Prev Policy.

[28] Lynch GS, Ryall JG (2008). Role of beta-adrenoceptor signaling in skeletal muscle: implications for muscle wasting and disease. Physiol Rev.

[29] Burniston JG, Ng Y, Clark WA, Colyer J, Tan LB, Goldspink DF (2002). Myotoxic effects of clenbuterol in the rat heart and soleus muscle. J Appl Physiol (1985).

[30] Shapland JE, Garner HE, Hatfield DG (1981). Cardiopulmonary effects of clenbuterol in the horse. J Vet Pharmacol Ther.

[31] Zeman RJ, Ludemann R, Easton TG, Etlinger JD (1988). Slow to fast alterations in skeletal muscle fibers caused by clenbuterol, a beta 2-receptor agonist. Am J Physiol.

[32] Maltin CA, Delday MI, Hay SM, Smith FG, Lobley GE, Reeds PJ (1987). The effect of the anabolic agent, clenbuterol, on overloaded rat skeletal muscle. Biosci Rep.

[33] Beekley MD, Ideus JM, Brechue WF, Kearns CF, McKeever KH (2003). Chronic clenbuterol administration alters myosin heavy chain composition in standardbred mares. Vet J.

[34] Kearns CF, McKeever KH (2002). Clenbuterol diminishes aerobic performance in horses. Med Sci Sports Exerc.

[35] Lynch GS, Hayes A, Campbell SP, Williams DA (1996). Effects of beta 2-agonist administration and exercise on contractile activation of skeletal muscle fibers. J Appl Physiol (1985).

[36] Plant DR, Kearns CF, McKeever KH, Lynch GS (2003). Therapeutic clenbuterol treatment does not alter Ca2+ sensitivity of permeabilized fast muscle fibres from exercise trained or untrained horses. J Muscle Res Cell Motil.

[37] Kearns CF, McKeever KH, Malinowski K, Struck MB, Abe T (2001). Chronic administration of therapeutic levels of clenbuterol acts as a repartitioning agent. J Appl Physiol (1985).

[38] Kearns CF, McKeever KH, Roegner V, Brady SM, Malinowski K (2006). Adiponectin and leptin are related to fat mass in horses. Vet J.

[39] (2025). Clenbuterol: what you need to know. https://www.fda.gov/animal-veterinary/animal-drug-safety/clenbuterol-what-you-need-know.

[40] Querin G, D'Ascenzo C, Peterle E (2013). Pilot trial of clenbuterol in spinal and bulbar muscular atrophy. Neurology.

[41] Mantovani G, Macciò A, Massa E, Madeddu C (2001). Managing cancer-related anorexia/cachexia. Drugs.

[42] Gorjao R, Dos Santos CM, Serdan TD (2019). New insights on the regulation of cancer cachexia by N-3 polyunsaturated fatty acids. Pharmacol Ther.

[43] Alhasso A, Glazener CM, Pickard R, N'dow J (2005). Adrenergic drugs for urinary incontinence in adults. Cochrane Database Syst Rev.

[44] Barrot M, Yalcin I, Choucair-Jaafar N, Benbouzid M, Freund-Mercier MJ (2009). From antidepressant drugs to beta-mimetics: preclinical insights on potential new treatments for neuropathic pain. Recent Pat CNS Drug Discov.

[45] Aggarwal A, Ansari AH, Isser HS, Kumar D (2025). Clenbuterol-induced myocardial infarction in a young bodybuilder. BMJ Case Rep.

[46] Kearns CF, McKeever KH (2009). Clenbuterol and the horse revisited. Vet J.

[47] Kuiper HA, Noordam MY, van Dooren-Flipsen MM, Schilt R, Roos AH (1998). Illegal use of beta-adrenergic agonists: European community. J Anim Sci.

[48] Yan H, Xu D, Meng H, Shi L, Li L (2015). Food poisoning by clenbuterol in China. Qual Assur Saf Crops Foods.

[49] (2016). Anabolic agents and meat contamination. https://www.usada.org/spirit-of-sport/clenbuterol-and-meat-contamination/.

[50] (2025). Alberto Contador stripped of Tour title. https://www.espn.in/olympics/cycling/story/_/id/7545798/alberto-contador-stripped-2010-tour-de-france-title.

[51] (2025). List of doping cases in sport by substance. https://en.m.wikipedia.org/w/index.php?oldid=1281908593&title=List_of_doping_cases_in_sport_by_substance.

